# Integration of Protein-Protein Interaction Networks and Gene Expression Profiles Helps Detect Pancreatic Adenocarcinoma Candidate Genes

**DOI:** 10.3389/fgene.2022.854661

**Published:** 2022-05-26

**Authors:** Lili Su, Guang Liu, Ying Guo, Xuanping Zhang, Xiaoyan Zhu, Jiayin Wang

**Affiliations:** ^1^ College of Electronics and Information Engineering, School of Computer Science and Technology, Xi’an Jiaotong University, Xi’an, China; ^2^ Department of Histology and Embryology, College of Basic Medical Sciences, Jilin University, Changchun, China

**Keywords:** cancer-associated genes, co-expression, protein-protein interaction, pancreatic adenocarcinoma, machine learning

## Abstract

More and more cancer-associated genes (CAGs) are being identified with the development of biological mechanism research. Integrative analysis of protein-protein interaction (PPI) networks and co-expression patterns of these genes can help identify new disease-associated genes and clarify their importance in specific diseases. This study proposed a PPI network and co-expression integration analysis model (PRNet) to integrate PPI networks and gene co-expression patterns to identify potential risk causative genes for pancreatic adenocarcinoma (PAAD). We scored the importance of the candidate genes by constructing a high-confidence co-expression-based edge-weighted PPI network, extracting protein regulatory sub-networks by random walk algorithm, constructing disease-specific networks based on known CAGs, and scoring the genes of the sub-networks with the PageRank algorithm. The results showed that our screened top-ranked genes were more critical in tumours relative to the known CAGs list and significantly differentiated the overall survival of PAAD patients. These results suggest that the PRNet method of ranking cancer-associated genes can identify new disease-associated genes and is more informative than the original CAGs list, which can help investigators to screen potential biomarkers for validation and molecular mechanism exploration.

## Introduction

Abnormal gene regulation and uncontrolled growth of cells in pancreatic tissue can lead to pancreatic cancer (McGuigan et al., 2018; Yao et al., 2020). Pancreatic adenocarcinoma (PAAD) accounts for approximately 80% or more of all types of pancreatic cancer and is the most common type of pancreatic cancer. This tumour is one of the most common cancers and is the top 10 leading causes of cancer-related death (Siegel et al., 2020). Therefore, it is critical to screen and identify potential biomarkers that can be used to treat PAAD, which will significantly improve the quality of life and prognosis of PAAD patients.

Tumour tissue is highly heterogeneous, and its complexity is a significant obstacle to a comprehensive understanding of the molecular mechanisms underlying tumour development (Qian et al., 2020). Researchers have uncovered potential risk-causing genes in tumour progression for many years (Bilici 2014; Le et al., 2016; Loosen et al., 2017). With advances in experimental tools and high-throughput sequencing technologies, an increasing number of genes are closely associated with cancer progression. The Cancer Gene Census (CGC) database (Sondka et al., 2018) screens candidate genes by searching for the presence of typical oncogene somatic mutation patterns, and determines the biological functions of candidate genes through literature curation, and has identified mutations affecting the molecular mechanism of gene dysfunction, which in turn explains the rationale for oncogenic transformation of the gene. The latest gene-wide screening described 719 genes that play essential functions in pan-cancer, providing researchers with great help in understanding cancer pathogenesis. However, whether these genes function in pancreatic adenocarcinoma tumour tissue and whether there are new pancreatic adenocarcinoma-specific biomarkers remain to be further investigated.

The development of computational methods for tumour candidate biomarker identification and ranking could fill the gap mentioned above. Alshahrani and Hoehndorf (2018) incorporated phenotypic similarity into network-based characterization learning and conveyed phenotypic association information through PPI networks to address the incompleteness of gene-phenotype knowledge. The features generated by their proposed SmuDGE algorithm can characterize gene-disease associations. By integrating information from various aspects such as PubMed abstracts, pathways, interactions, gene ontology, disease ontology, sequence similarity, and constructing bayesian ridge regression models, the pBRIT developed by Kumar et al. (2018) can prioritize disease genes. Recently, Li et al. (2019) constructed a graph convolutional neural network-based gene ranking algorithm PGCN by training both embedded learning models and association prediction models in an end-to-end manner and discovered some disease-associated candidate genes. These approaches have achieved a great success in exploring potential disease-associated genes, which also suggest that integrating multifaceted biological information can help us better delineate the gene-disease associations and thus screen key candidate genes. The PageRank algorithm is one of the most widely used page ranking algorithms, it is developed by Google and is named after Larry Page, Google’s co-founder and president (Brin et al., 1998). When applied to biological networks, PageRank has a great stability can help evaluate important nodes and pathways in directed networks, such as metabolic networks (Iván and Grolmusz 2011).

With the development of high-throughput sequencing technology, tumour-related sequencing profiles are increasingly accumulated. Bioinformatics analysis based on expression profiling data has emerged in studying the molecular mechanisms of tumorigenesis and development. This study proposed a PPI network and co-expression integration analysis model (PRNet) to integrate PPI network and gene co-expression patterns to identify potential risk causative genes in pancreatic adenocarcinoma (PAAD). We scored the importance of candidate genes by PRNet to screen new key regulators based on existing cancer-related genes and PageRank algorithm is used for network scoring in PRNet. Moreover, the reliability of our ranked candidate genes was further validated by various analytical tools. Overall, our data can give a guide to the study of the biological mechanisms of PAAD development and provide new insights for the future treatment of PAAD.

## Materials and Methods

### Dataset Preparation

TCGA PAAD transcriptome normalization data and clinical information were downloaded from UCSC Xena (https://xenabrowser.net/datapages/) for further co-expression analysis. The list of cancer-associated genes was obtained from the Cancer Gene Census (https://cancer.sanger.ac.uk/census). CERES-dependent scores were based on data from the Crispr technology to knockdown target genes and perform cell depletion assays (Ghandi et al., 2019). Target gene dependency scores were obtained from the DepMap web portal (https://depmap.org/portal/download/). A lower CERES score for a given gene in a given cell line indicates that the gene of interest plays an essential function in that cell line. A score of 0 indicates that the gene is not essential; accordingly, a score of -1 is the median of all pan-essential gene function scores. The experimentally validated protein interaction information was obtained by integrating BioGRID (Merriel et al., 2011), I2D (Brown and Jurisica 2007), BioPlex (Huttlin et al., 2017) and IntAct (Hermjakob et al., 2004).

### Weighting Protein-Protein Interactions

Based on the TCGA PAAD transcriptome data, we can analyze the co-expression relationship between genes. The stronger co-expression relationship indicates that the two relationships are more functionally related. We first calculated the correlations of all possible gene pairs and filtered them based on the PPI network, keeping only the gene pairs with PPI present. Thus, the weight between each gene pair can be expressed as.

$$W_{\left(A,B\right)}=\{\begin{array}{c} corr\left(A,\;B\right),\;\;\;Pair\left(A,\;B\right)=1\\ NA,\;\;\;\;\;\;\;\;\;\;\;\;\;\;\;\;\;Pair\left(A,\;B\right)=0 \end{array}$$

Where $W_{\left(A,\;B\right)}$ denotes the weight of the linkage between gene A and gene B. Pair(A, B)=1 indicates the presence of interaction between gene A and gene B, while the opposite does not exist. We only keep the gene pairs with interactions to calculate their weights. corr(A, B) indicates the correlation between the expression levels of gene A and gene B.

### Generating Disease-Specific Networks

After weighting the PPI network, we use the short random walks algorithm (Barnes and Feige, 1993) to decompose the network into a series of sub-networks. This step is implemented based on the cluster_walktrap function of the R package igraph. The core idea is that short random walks are more likely to be enriched in the same community. Therefore, we filtered the subnetworks based on the collected CAGs and only retained subnetworks containing at least one CAG for subsequent analysis. This means that PRNet filters sub-networks containing at least one CAG instead of the entire weighted PPT network to construct disease-specific networks, which can better retain disease-specific information.

### Ranking Candidate Genes

PRNet uses the PageRank algorithm (Xing and Ghorbani, 2004) to calculate all the final filtered sub-networks, which returns the PageRank value of each node, which is considered the importance level of the gene. The specific algorithm is as follows.

$$PR\left(gene_{i}\right)=\frac{1-q}{N}+q\overset{k}{\underset{gene_{j}}{\sum }}\left(\frac{PR\left(gene_{j}\right)}{L\left(gene_{j}\right)}\right)$$

Where $gene_{i}$ is the genes to be studied, and $gene_{j}$ stand for the genes interacting with $gene_{i}$ . k is the number of genes interacting with the gene to be studied, $PR\left(gene_{i}\right)$ is the PageRank value of the gene, $L\left(gene_{j}\right)$ is the number of genes chained from $gene_{j}$ . The ‘N’ means the total amount of genes used for analysis, and the ‘q’ is the damping factor, the meaning of which is the probability of reaching a gene and continues to navigate backwards. The default value of ‘q’ is 0.85.

Based on this, we can calculate the PageRank value of each gene and use it to indicate the relative importance of the gene in the disease-specific network and then potential screen biomarkers.

### Bioinformatics Analysis

Based on the TCGA PAAD gene expression normalized count matrix, we divided the samples into two subgroups by unsupervised hierarchical clustering using the R package “ConsensusClusterPlus” (Wilkerson and Hayes 2010). Then the matrix was processed as log2 (FPKM +1) and genes with zero expression in more than half of the samples were removed. The DESeq2 package (Love et al., 2014) was used to calculate the differential expression of all genes between the different groups, where adjusted *p*-values less than 0.05 and | log2FoldChange|>1 were considered differentially expressed genes. Principal component analysis (PCA) (Wold et al., 1987) revealed differences in expression patterns between different subgroups of PAAD. Gene set enrichment analysis (GSEA) (Shi and Walker 2007) was used to perform enrichment analysis of differential expression data. False discovery rate (FDR) q-value <0.05 was set as the cut-off criterion. Based on the Kyoto Encyclopedia of Genes and Genomes (KEGG) signaling pathway, we performed gene set enrichment analysis using the R package “clusterProfiler” v3.18.1. The enzyme, transcription factor, FDA approved drugs information were downloaded from the Human Protein Atlas (HPA) database. Survival analysis was performed using the R package “survival,” and the overall survival curve was obtained by Kaplan-Meier estimation.

## Results

### Prioritizing Disease Candidate Genes

**FIGURE 1:**
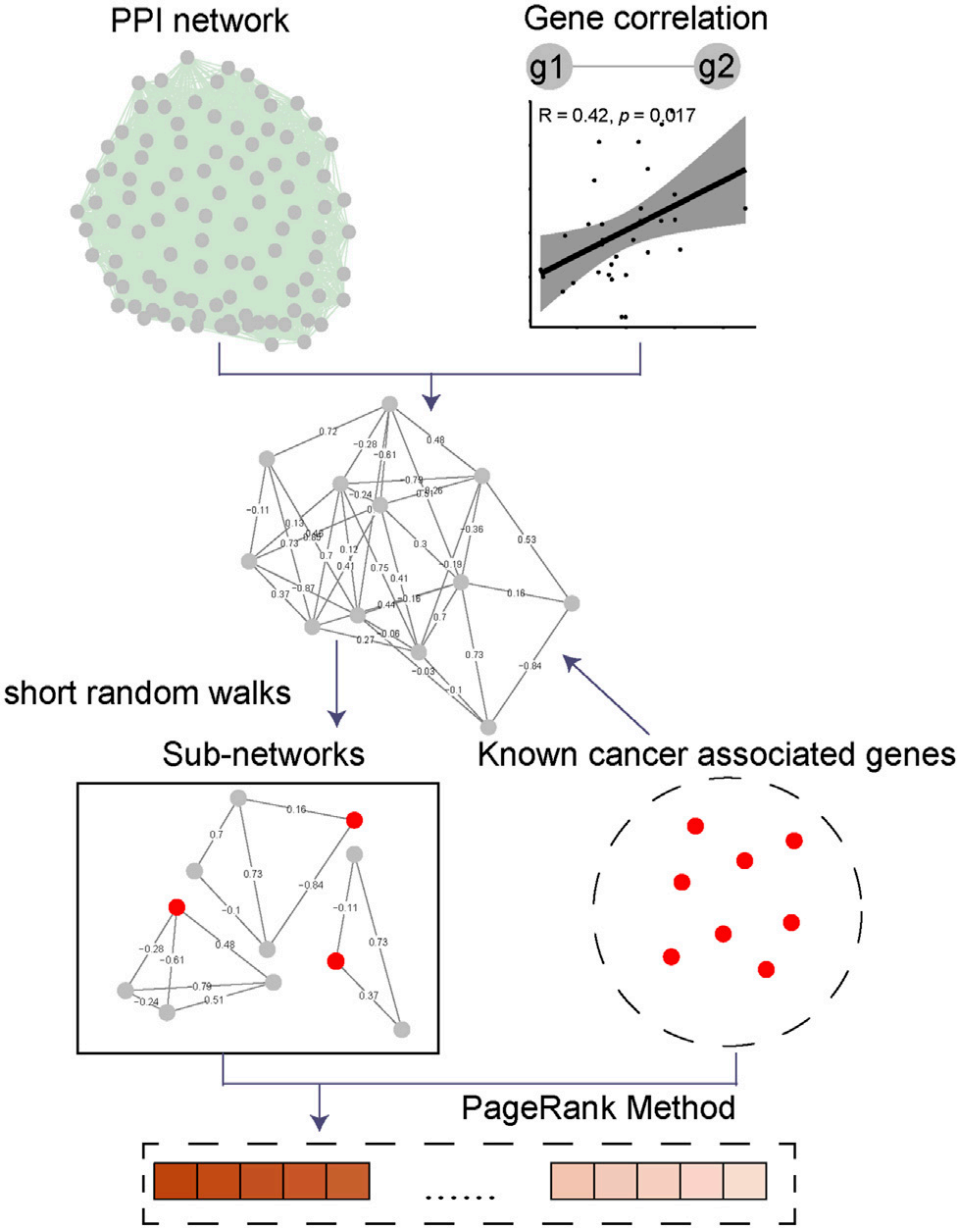
Flowchart of PRNet. The protein interaction information from validated PPI databases. Gene pairs’ expression correlation was calculated by using TCGA PAAD mRNA expression matrix. These datasets were merged to construct a standard PAAD-specific weighted-PPI network. Short random walks algorithm was used to slice this extensive network into multiple communities, and kept only the communities containing known CAGs as disease-specific sub-networks. Finally, the PageRank algorithm was applied to analyze the final constructed network and calculated the PR value of each node.

The basic flow of model construction is shown in Figure 1. We curated the PPI information from several databases including 204,399 interactions from BioGRID, 16,334 interactions from I2D (Brown and Jurisica 2007), 28,382 interactions from BioPlex (Huttlin et al., 2017) and 109,220 interactions from IntAct (Hermjakob et al., 2004). These protein interactions collected were all experimentally validated with high confidence, so we directly merged the results from all databases. A total of 358,335 interactions among 20,379 proteins were finally collected, and after removing duplicate protein interaction pairs, 309,321 experimentally identified interactions among 20,379 proteins were obtained for further analysis. Next, we downloaded TCGA PAAD transcriptome data, calculated gene pairs’ expression correlation in the human-derived PPI network. We merged the correlation coefficients into the PPI network as the weights of gene pairs to construct a standard PAAD-specific weighted-PPI network. Next, we analyzed this network using short random walks, sliced this extensive network into multiple communities, and kept only the communities containing known CAGs as disease-specific sub-networks. A total of 147 communities were generated as a result and only 19 communities contain the known CAGs were obtained for further analysis. We used the PageRank algorithm to analyze the final constructed network and calculated the PR value of each node. Higher PR values indicate that the gene plays a more regulatory role in PAAD, i.e., more important for PAAD development. Lower PR values indicate that the gene is more isolated in the gene regulatory network of PAAD and less likely to act as a candidate in PAAD.

### Novel PAAD Candidate Genes

Based on the above method, we calculated the PR values of 14,615 genes in PAAD, and the ranking and PR values of these genes were shown in Supplementary Table S1. We selected three subnetworks containing the most genes and performed KEGG enrichment analysis (Supplementary Figure S1 and Supplementary Table S2). The largest subnetwork contains many pathways related to cell proliferation and division, and maintenance of life activities such as cell cycle, DNA replication, ubiquitin-mediated proteolysis, etc. The pathways involved in the second subnetwork are related to malignant tumour progression, such as MAPK signaling pathway, ERBB signaling pathway, VEGF signaling pathway, etc. The third major sub-network is related to ribosomes.

**FIGURE 2:**
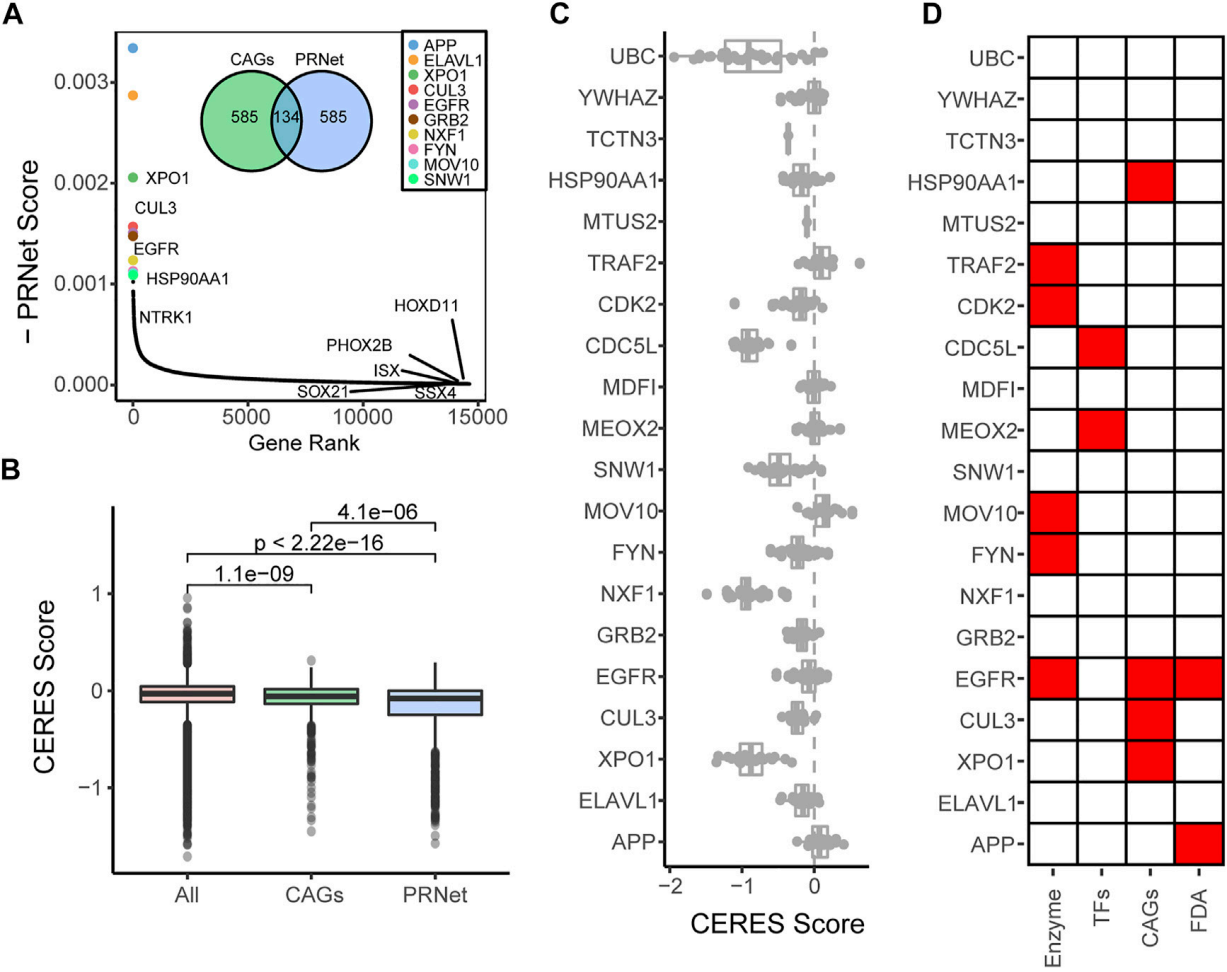
Novel PAAD candidates’ features. **(A)** The distribution of the known CAGs after reordering. **(B)** The CERES scores of the top 719 genes and the CAG list relative to all genes. **(C)** The CERES score distribution of the 20 top-ranked genes. **(D)** Functional annotation of the 20 top-ranked genes.

**FIGURE 3:**
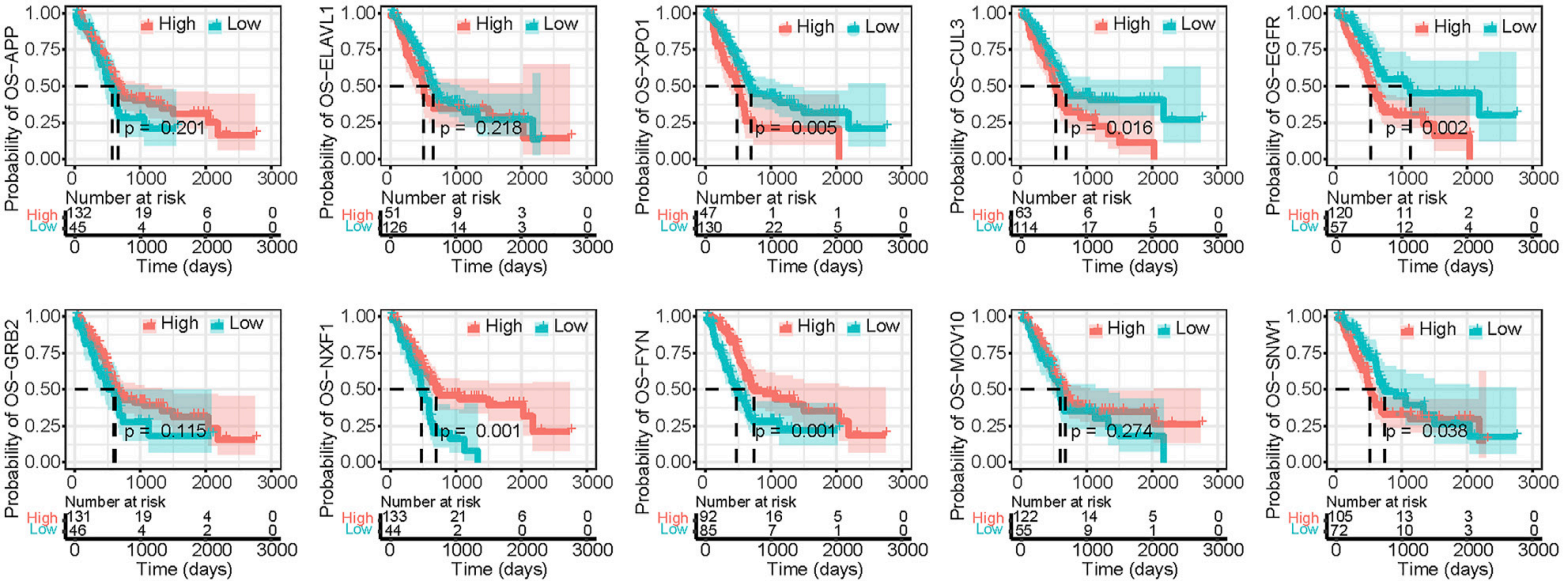
Kaplan–Meier OS curves of genes ranked from 1 to 10.

We further compared the distribution of the known CAGs after reordering. As shown in Figure 2A, there are XX identical genes in the top 719 genes and the CAG list, XPO1, CUL3, EGFR, HSP90AA1, NTRK1, etc., were clearly ranked at the top, further indicating their essential functions in the malignant progression PAAD. However, we also found that some CAGs such as HOXD11, PHOX2B, SSX4, ISX, SOX21 ranked significantly lower. Our selected CAG list is all about pan-cancer markers, but not all markers play essential functions in PAAD. To further confirm the reliability of our screening results, we analyzed the CERES scores of the top 719 genes and the CAG list relative to all genes (Figure 2B). The results showed that the CERES scores of PRNet screened genes were significantly lower than those of the known CAG list, indicating that these genes play more critical functions in PAAD. Then, we showed the top 20 genes, plotted their CERES score distribution individually, and annotated their functions (Figures 2C,D). Some genes are known as enzymes, transcription factors or tumour therapeutic targets. We checked the genetic alterations of the top 20 genes in TCGA PAAD cohorts and less mutations were found, in addition to the fact that most of the genes had samples that showed amplification in samples, especially YWHAZ, with a high frequency (7%) (Supplementary Figure S2A). Further functional enrichment showed that these genes were significantly enriched in some pathways which play important roles in PAAD progression, such as PI3K/AKT/mTOR signaling pathway (Kennedy et al., 2011), EGFR signaling pathway (Williams et al., 2017) and NF-κB signaling pathway (Prabhu et al., 2014), etc. (Supplementary Figure S2B). Survival analysis showed that the expression of most genes (12 of 20) correlated with the survival of PAAD patients (Figure 3 and Supplementary Figure S2C). However, many genes are less studied, which means their functions in PAAD deserve more exploration.

### Comparison of Top-Ranked Genes and CAGs

**FIGURE 4:**
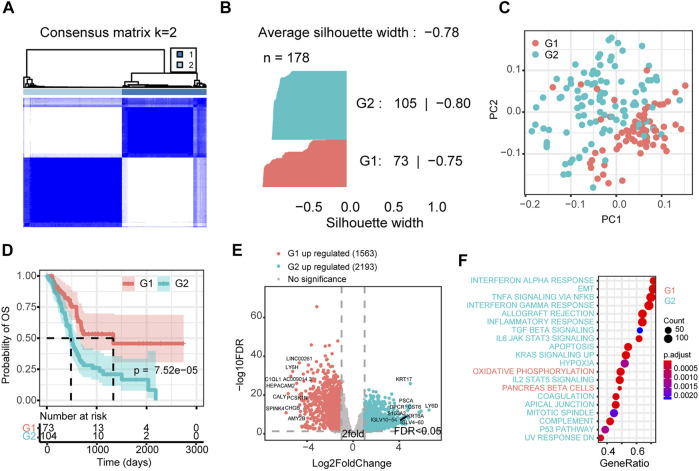
Consensus cluster of PAAD samples based on top-ranked genes. **(A)** Consensus cluster heatmap of PAAD samples. **(B)** The silhouette plot of the two clusters (G1/2) defined by the top-ranked genes. **(C)** Principal component analysis of the total mRNA expression profile in the TCGA dataset. **(D)** Kaplan–Meier OS curves for different subgroups. **(E)** Differentially expressed genes between G1 and G2 subgroup. **(F)** GSEA analysis of differentially expressed genes between G1 and G2 subgroup by using hallmark pathways. G2: high-risk subgroup; G1: low-risk subgroup.

To further elaborate the superiority of our screened model in PAAD, we selected the top 719 genes and CAG list for unsupervised hierarchical clustering (Figure 4A and Supplementary Figure S3A). For comparison, we defined both as two classes (Supplementary Table S3). Compared with the CAG list derived classification system, the classification system of the PRNet-based signature definition was significantly better with a silhouette width of −0.78 (Figure 4B and Supplementary Figure S3B). Although there were significant transcriptome differences in both signature-defined subgroups (Figure 4C and Supplementary Figure S3C), interestingly, the subgroup obtained based on the CAG list did not suggest survival differences (Supplementary Figure S3D). The subgroups classified based on the PRNet-defined signature could significantly distinguish survival (Figure 4D). We further investigated the differences in gene expression and pathway activity between these two groups. As shown in Figure 4E, 1,563 genes were significantly overexpressed in the low-risk group, and 2,193 genes were significantly elevated in the high-risk group (Supplementary Table S4). We then analyzed the pathways affected by these genes. Tumour hallmark signaling pathway enrichment analysis suggested that the high-risk group was significantly enriched in interferon response, epithelial-mesenchymal transition, TGFβ and other immune response and oncogenic pathways. In contrast, the low-risk group showed normal pancreatic cells’ molecular characteristics, overexpressed some markers of normal pancreatic cells, and showed higher oxidative phosphorylation and ribosomal signaling pathway activities (Figure 4F, Supplementary Figure S4 and Supplementary Table S5).

We also compared the association of high and low-risk groups with clinical factors to investigate a significant survival difference between the two groups (Supplementary Figure S5). Interestingly, no significant differences were found at either the age or sex level. However, there were more samples of lymph node metastases and distal metastases in the high-risk group, and they were more enriched in stage III and stage IV tumours. This corresponds to a worse prognosis for survival at later clinical stages. The worse overall survival in the high-risk group compared to the low-risk group may be related to their greater susceptibility to lymph node metastasis and distal metastasis.

## Discussion

Pancreatic adenocarcinoma is a kind of tumour of highly heterogeneous, which leads a big issue for researchers to get a comprehensive understanding in the study of tumour molecular mechanisms (McGuigan et al., 2018; Yao et al., 2020). Attempts have been made for many years to discover the potential risk-causing genes working in PAAD progression. In our study, an analysis model integrating PPI network and co-expression patterns is proposed, which can identify potential risk causative genes in pancreatic adenocarcinoma. The importance of candidate genes was scored by PRNet to screen new pivotal regulators. Furthermore, the reliability of the screened candidate genes was validated by kinds of analytical tools.

Based on the PRNet method, we constructed the disease-specific subnetworks. As shown in Supplementary Figure S1 and Supplementary Table S2, pathways related to cell cycle and life activity maintenance are mainly in the subnetwork with the most significant number of genes. And pathways related to tumour progression, such as MAPK and ERBB signaling pathway, etc., presents in the second-largest subnetwork. These results indicate that the networks we screened are closely related to tumours. Cancer is characterized by uncontrolled cell proliferation, which results from the abnormal expression and activities of various cell cycle proteins, making the cell cycle a typical hotspot in tumour research (Sherr 1996). In addition, MAPK, VEGF and other signaling pathways have been proved closely associated with malignant tumour progression in many studies (Ferrara 2005; Wagner and Nebreda 2009; Gargalionis et al., 2018). Therefore, analysis based on these networks may help identify core regulatory proteins and explore their potential as tumour biomarkers.

Looking into the top 20 rank score potential biomarkers in Figure 2, some of them have been proved the functions in PAAD, such as XPO1 and FYN. XPO1 mRNA expression is heterogeneous in pancreatic adenocarcinoma and is associated with progression stage and shorter survival (Birnbaum et al., 2019). Nuclear export Inhibitors based on study of XPO1 are used to therapy pancreatic cancer (Muqbil et al. 2018). FYN is one of Src family kinases. Its upregulation is associated with pancreatic cancer metastasis (Chen et al., 2010). Interestingly, our study found some proteins that are not annotated as enzymes, transcription factors, and FDA-validated targets, such as APP, ELAVL1 and YWHAZ. As a potential biomarker with the highest PageRank scores, amyloid precursor protein (APP) is a substrate of proteases and secretase, which is mainly focused on pathogenesis of Alzheimer’s disease, was reported been involved in pancreatic cancer (Hansel et al., 2003). And further study figured out that the major enzyme mediating APP cleavage in pancreatic cancer is ASAM10 (Woods and Padmanabhan 2013). It has been suggested that ELAVL1 is an RNA-binding protein, which may play a translational modification regulatory sway in prostate cancer progression (Melling et al., 2016). In addition, ELAVL1 may influence the efficacy of glioma heterogeneous targeted drugs by affecting cell fusion (Filippova and Nabors 2020). ELAVL1 also plays an essential function in tumours such as colorectal cancer (Gu et al., 2019) and breast cancer (Chou et al., 2015; Luo et al., 2020), suggesting that ELAVL1 may indeed serve as a potential marker for PAAD. In contrast, YWHAZ, despite its low gene expression and not being easily detectable, is involved in tumorigenesis and progression in many tumours (Nishimura et al., 2013; Guo et al., 2018; Zhao et al., 2018), including pancreatic cancer (Xue et al., 2018). These results suggest that the potential genes we screened effectively-identified novel markers not found in the standard gene set.

Overall, our proposed model is superior in reconstructing potential tumour markers and identifying new therapeutic targets and revealing more tumour heterogeneity than the traditional CAG list. These results offer a way to study the pancreatic adenocarcinoma’s pathogenesis and provide new ideas for the future drug development and clinical treatment of pancreatic adenocarcinoma.

## Data Availability

Publicly available datasets were analyzed in this study. This data can be found here: The Cancer Genome Atlas (TCGA), Datasets link: https://xenabrowser.net/datapages/.
