# Supplementary figures and images for: Integration of Protein-Protein Interaction Networks and Gene Expression Profiles Helps Detect Pancreatic Adenocarcinoma Candidate Genes
Source: Front Genet. 2022 May 26;13:854661. doi: 10.3389/fgene.2022.854661 (PMC9197464; doi:10.3389/fgene.2022.854661)

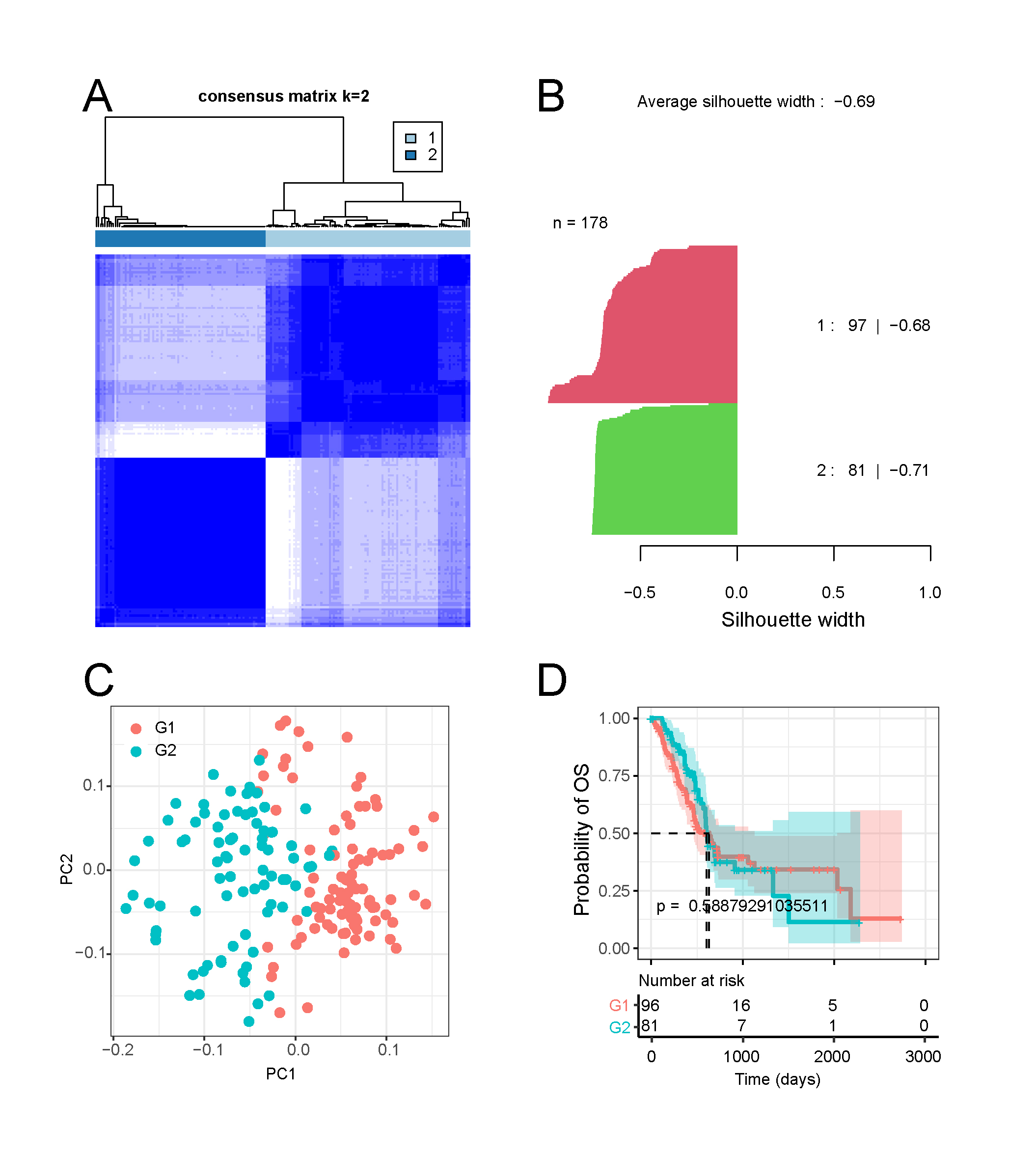

Supplement: Supplementary file 2 [file Image3.JPEG]

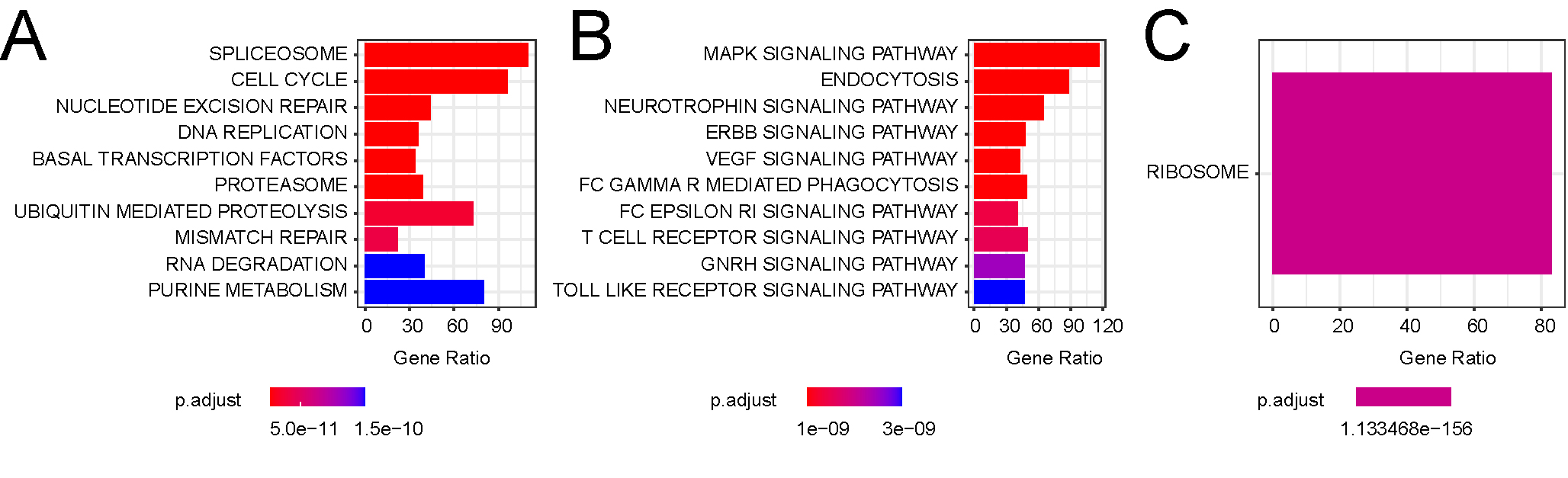

Supplement: Supplementary file 4 [file Image1.JPEG]

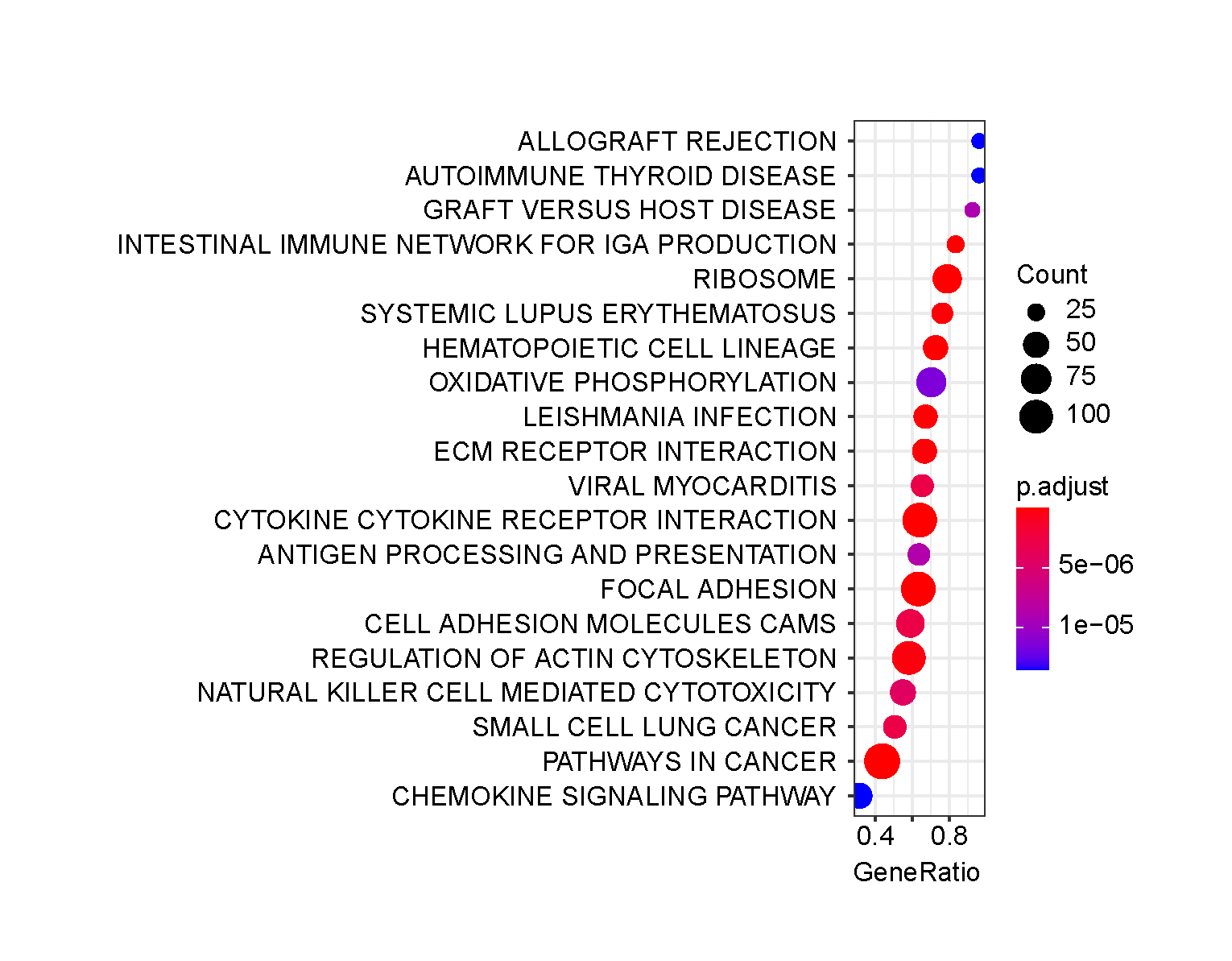

Supplement: Supplementary file 5 [file Image4.JPEG]

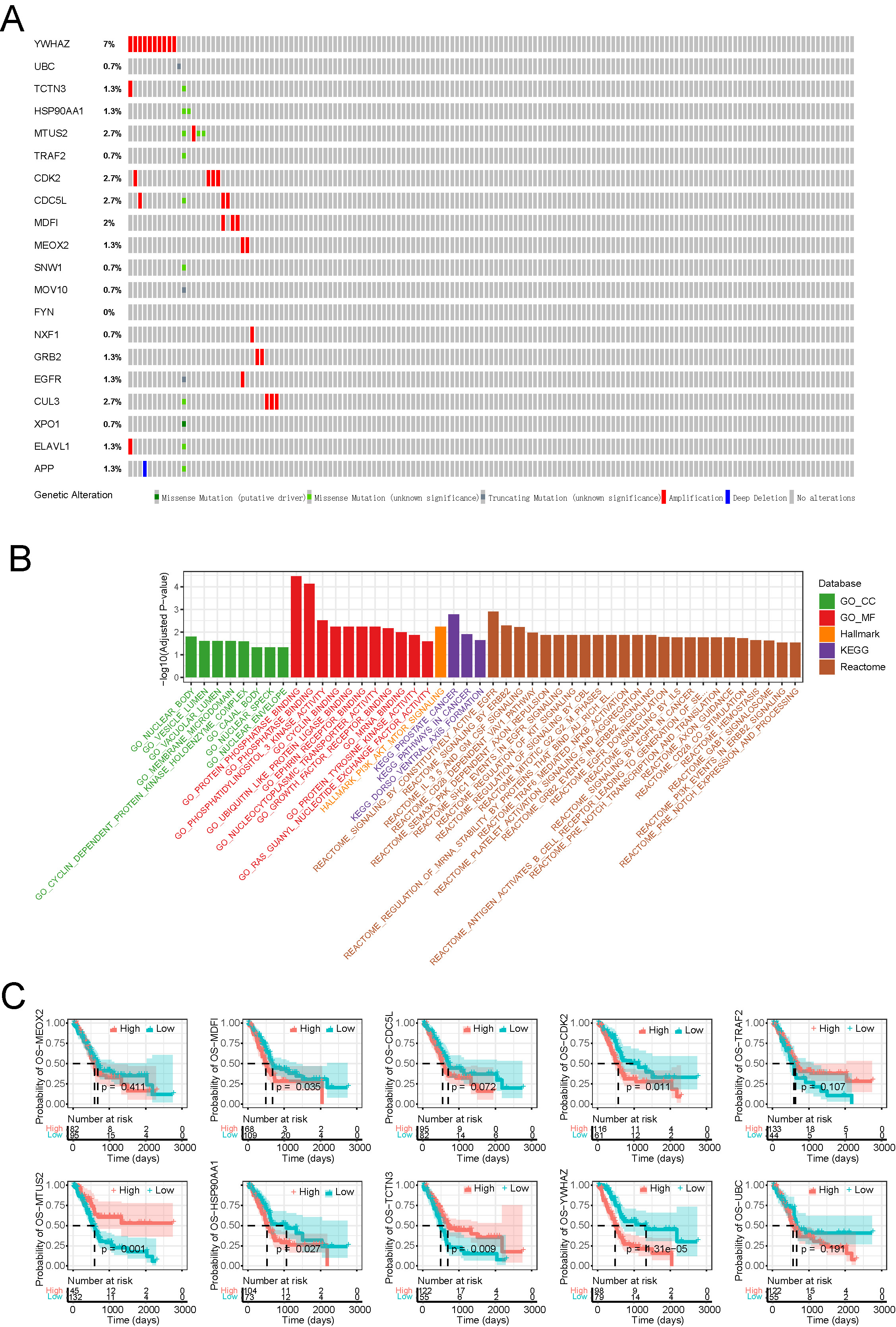

Supplement: Supplementary file 6 [file Image2.JPEG]

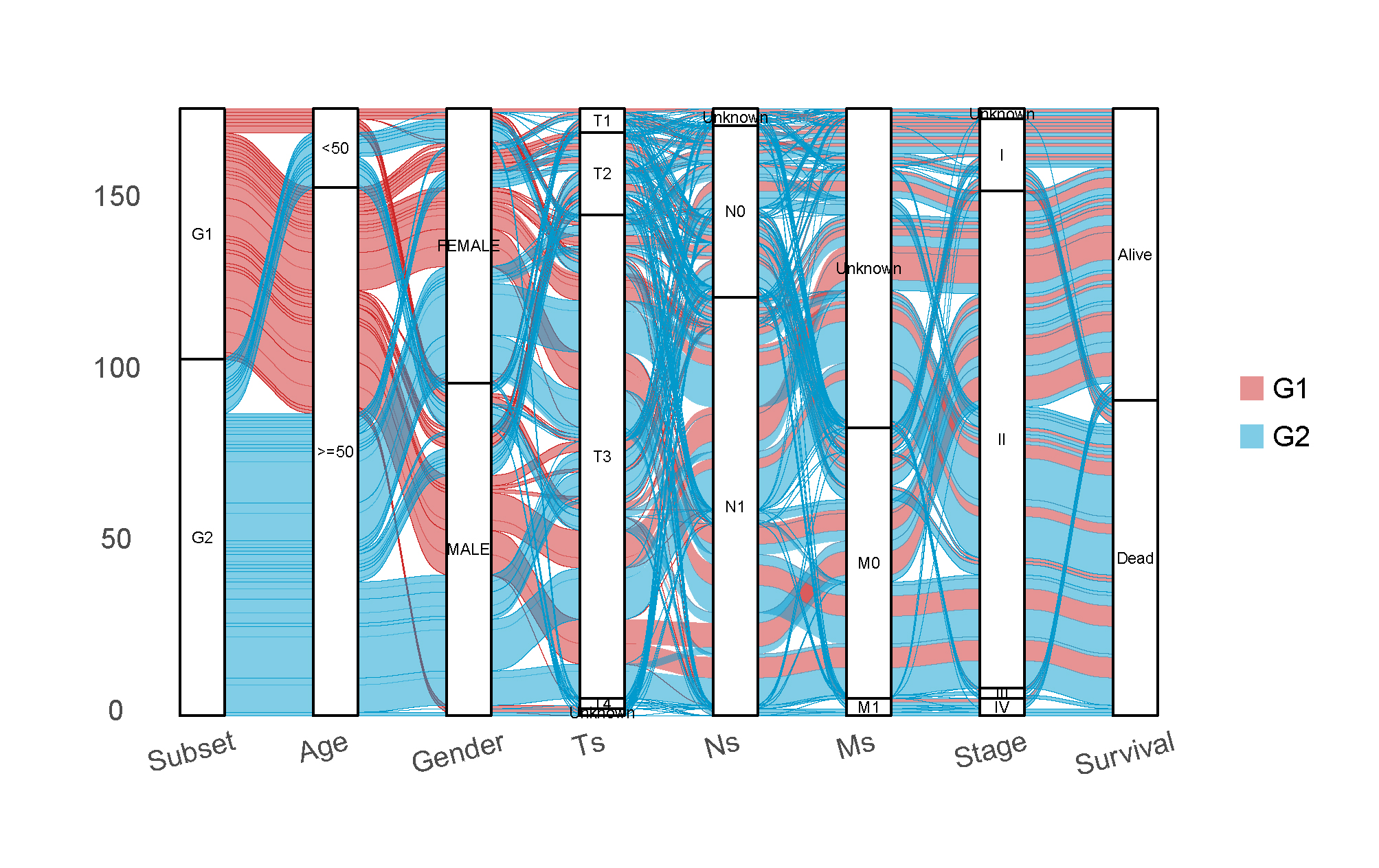

Supplement: Supplementary file 7 [file Image5.JPEG]
