# Supplementary material for: Integration of Protein-Protein Interaction Networks and Gene Expression Profiles Helps Detect Pancreatic Adenocarcinoma Candidate Genes
Source: Front Genet. 2022 May 26;13:854661. doi: 10.3389/fgene.2022.854661 (PMC9197464; doi:10.3389/fgene.2022.854661)
Supplement: Supplementary file 11 [file DataSheet1.docx]

**Supporting Information**

**Figure S1.** KEGG pathway enrichment analysis of top three largest subnetworks. (A) KEGG pathway enrichment analysis of the largest subnetworks. (B) KEGG pathway enrichment analysis of the second-largest subnetworks. (C) KEGG pathway enrichment analysis of the third-largest subnetworks.

**Figure S2.** Clinical significance of top 20 PRNet screened genes alterations in patients with PAAD. (A) Genetic alteration profiles of the genes in TCGA PAAD cohort. (B)Functional enrichment analysis. (C) Kaplan–Meier OS curves of genes ranked from 11 to 20.

**Figure S3.** Consensus cluster of PAAD samples based on CAGs. (A) Consensus cluster heatmap of PAAD samples. (B) The silhouette plot of the two clusters (G1/2) defined by the CAGs. (C) Principal component analysis of the total mRNA expression profile in the TCGA dataset. (D) Kaplan–Meier OS curves for different subgroups.

**Figure S4.** GSEA analysis of differentially expressed genes between G1 and G2 subgroup by using KEGG pathways.

**Figure S5.** Sankey plot of clusters and clinical information.

**Table S1.** Gene ranks with the PRNet algorithm.

**Table S2.** KEGG pathway enrichment analysis of top three largest subnetworks.

**Table S3.** Sample groups after unsupervised consensus clustering by using CAG list and PRNet genes.

**Table S4.** Differentially expressed genes between G1 and G2 subgroup.

**Table S5.** GSEA analysis of differentially expressed genes between G1 and G2 subgroup by using hallmark and KEGG pathways.
